# Use of the Ion PGM and the GeneReader NGS Systems in Daily Routine Practice for Advanced Lung Adenocarcinoma Patients: A Practical Point of View Reporting a Comparative Study and Assessment of 90 Patients

**DOI:** 10.3390/cancers10040088

**Published:** 2018-03-21

**Authors:** Simon Heeke, Véronique Hofman, Elodie Long-Mira, Virginie Lespinet, Salomé Lalvée, Olivier Bordone, Camille Ribeyre, Virginie Tanga, Jonathan Benzaquen, Sylvie Leroy, Charlotte Cohen, Jérôme Mouroux, Charles Hugo Marquette, Marius Ilié, Paul Hofman

**Affiliations:** 1 Team 4, Institute of Research on Cancer and Aging of Nice (IRCAN), Inserm U1081, CNRS UMR7284, Université Côte d’Azur, CHU de Nice, 06107 Nice Cedex 2, France; heeke.s@chu-nice.fr (S.H.); HOFMAN.V@chu-nice.fr (V.H.); long-mira.e@chu-nice.fr (E.L.-M.); benzaquen.j@chu-nice.fr (J.B.); marquette.c@chu-nice.fr (C.H.M.); ilie.m@chu-nice.fr (M.I.); 2 Laboratory of Clinical and Experimental Pathology, Université Côte d’Azur, CHU de Nice, University Hospital Federation OncoAge, 06001 Nice Cedex 1, France; LESPINET-FABRE.V@chu-nice.fr (V.L.); lalvee.s@chu-nice.fr (S.L.); bordone.o@chu-nice.fr (O.B.); ribeyre.camille@gmail.com (C.R.); TANGA.V@chu-nice.fr (V.T.); 3 Hospital-Integrated Biobank (BB-0033-00025), Université Côte d’Azur, CHU de Nice, University Hospital Federation OncoAge, 06001 Nice Cedex 1, France; 4 Department of Pulmonary Medicine and Oncology, Université Côte d’Azur, CHU de Nice, University Hospital Federation OncoAge, 06001 Nice Cedex 1, France; leroy.s2@chu-nice.fr; 5 Department of Thoracic Surgery, Université Côte d’Azur, CHU de Nice, University Hospital Federation OncoAge, 06001 Nice Cedex 1, France; cohen.c@chu-nice.fr (C.C.); mouroux.j@chu-nice.fr (J.M.)

**Keywords:** next-generation sequencing, lung adenocarcinoma, molecular pathology, Ion PGM, GeneReader

## Abstract

**Background**: With the integration of various targeted therapies into the clinical management of patients with advanced lung adenocarcinoma, next-generation sequencing (NGS) has become the technology of choice and has led to an increase in simultaneously interrogated genes. However, the broader adoption of NGS for routine clinical practice is still hampered by sophisticated workflows, complex bioinformatics analysis and medical interpretation. Therefore, the performance of the novel QIAGEN GeneReader NGS system was compared to an in-house ISO-15189 certified Ion PGM NGS platform. **Methods**: Clinical samples from 90 patients (60 Retrospectively and 30 Prospectively) with lung adenocarcinoma were sequenced with both systems. Mutations were analyzed and *EGFR*, *KRAS*, *BRAF*, *NRAS*, *ALK*, *PIK3CA* and *ERBB2* genes were compared and sampling time and suitability for clinical testing were assessed. **Results**: Both sequencing systems showed perfect concordance for the overlapping genes. Correlation of allele frequency was *r*^2^ = 0.93 for the retrospective patients and *r*^2^ = 0.81 for the prospective patients. Hands-on time and total run time were shorter using the PGM system, while the GeneReader platform provided good traceability and up-to-date interpretation of the results. **Conclusion**: We demonstrated the suitability of the GeneReader NGS system in routine practice in a clinical pathology laboratory setting.

## 1. Introduction

The introduction of targeted therapies into the clinical management of lung adenocarcinoma has had a massive impact on patient care [1,2,3]. Multiple driver mutations are now druggable and treatments differ depending on the mutational pattern of each patient with advanced lung adenocarcinoma [4]. Consequently, mutational analysis is critical for treatment decisions in clinical routine practice. While several techniques have been developed and introduced into clinical care over the past few years, next-generation sequencing (NGS) has evolved to become the new standard in molecular diagnostics for most institutions [5,6]. Thus, the development of targeted sequencing panels, which only cover specific and relevant mutations, make NGS affordable and suitable for high-throughput processing. Two different sequencing techniques were established initially for NGS in clinics, the Ion Personal Genome Machine (PGM) and Illumina sequencing [7,8,9,10]. While both systems are based on fundamentally different sequencing principles, their purpose for clinical mutation analysis is quite similar. However, both systems require each diagnostic laboratory to develop their own workflow, leading to a plethora of “homebrew” solutions as different devices for sample preparation can be integrated in the respective workflows. Furthermore, there is no consensus system for individual sample tracking and the storage of meta information which is therefore required to be set up in each diagnostic laboratory individually. Additionally, setting up a NGS workflow requires well-trained personal, complex workflows and the need for specialized bioinformatics to analyze and interpret the results. Furthermore, the initial costs are relatively high in comparison to other targeted sequencing methods [11]. To overcome this problematic, Qiagen (Hilden, Germany) developed a novel sequencing system, the GeneReader, specifically designed for the needs of routine molecular diagnostics. In contrast to the existing solutions, the GeneReader system provides, in theory, a complete workflow from sample preparation to data analysis. This should decrease hands-on time during sample preparation, as well as increase traceability and reproducibility. Furthermore, bioinformatic analysis is integrated into the workflow and automated to further improve the quality and efficacy. All related information, like lot numbers, qualitative and quantitative information, as well as results are stored in a central software platform making the use of spreadsheets or other complicated solutions expendable. Additionally, it is offered in a pay-per-sample model, where initial costs are low and are incurred only for each sample tested. 

To analyze whether this approach has the potential to be adopted in a molecular pathology laboratory setting, we compared the performance of the GeneReader system with our current workflow, which is based on the Ion PGM system, accredited according to ISO 15189 (COFRAC Accreditation n° 8-3034) [12], and has been established in the Laboratory of Clinical and Experimental Pathology (LPCE, Centre Hospitalier Universitaire (CHU) Nice, Nice, France). 

The focus of our investigation was the concordance in mutation analysis, the usability of the two systems, as well as the time needed for sample preparation and data analysis. Therefore, we tested frozen or formalin-fixed paraffin-embedded (FFPE) samples from 90 patients with lung adenocarcinoma. A training set of 60 samples, previously characterized on the Ion PGM system, was retrospectively evaluated on the GeneReader. A validation set of 30 samples was prospectively tested in parallel on both systems in the clinical setting. As the sequencing panels from the Ion PGM and the Qiagen GeneReader covered different amplicons, we focused on the actionable overlapping genes, *EGFR*, *KRAS*, *BRAF*, *NRAS*, *ALK*, *PIK3CA* and *ERBB2* [13].

## 2. Results

### 2.1. GeneReader Reveals High Concordance with Ion PGM

As both sequencing systems use different amplicon panels (Table 1), we focused our comparative analysis on genes that are covered by both panels. 

In the retrospective patient cohort, 58 out of 60 (97%) patients were sequenced successfully on both systems, while for each of the respective sequencing systems, one sample failed sequencing due to failed library preparation. For the 58 successfully sequenced samples in the enriched retrospective cohort, 33 (55%) harbored mutations in the analyzed genes (Table 2). 

In the prospective cohort, mutations in the selected genes could be detected in 25 out of the 30 (83%) patients. Mutations were only detected in *EGFR*, *KRAS* and *BRAF* genes (Table 2, Supplementary Materials Table S1). Importantly, both systems showed 100% concordance for the overlapping genes. No false positive or false negative variants were detected on the GeneReader when compared to the Ion PGM (Supplementary Materials Table S2). However, for the prospective cohort, limited tumor material for one patient obliged the use of two different FFPE blocks for the DNA extraction. Subsequently, an *EGFR* mutation (p.G719C; c.2155G>T) was detected using the Ion PGM workflow, while another *EGFR* mutation (p.L858R; c.2573T>G) was diagnosed using the GeneReader platform. To exclude any technical errors for this discordant result, we tested the isolated DNA from the Ion PGM extraction on the GeneReader and vice versa. The initially tested mutations were confirmed by the second sequencing, demonstrating indeed their presence in the respective tumor samples. 

### 2.2. Allele Frequency between the Sequencing Systems Is Highly Correlated

**Figure 1:**
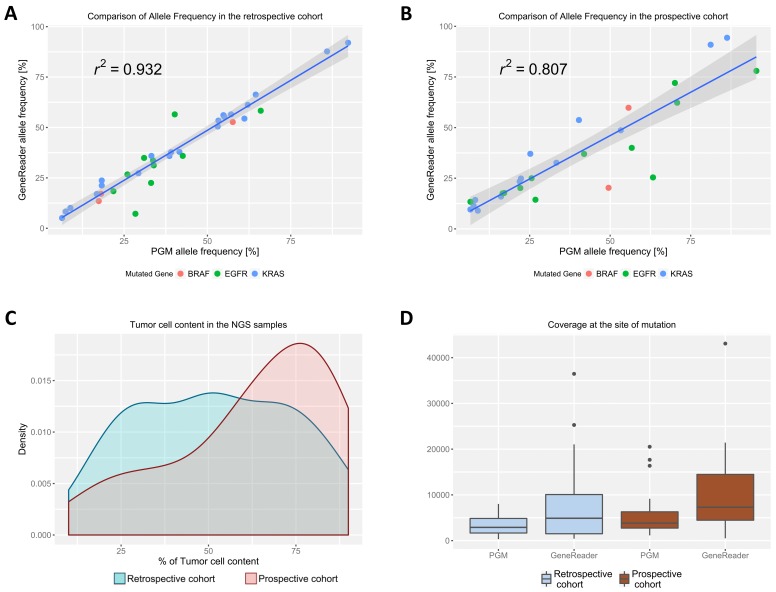
Correlation of the allele frequency for all mutations found between the GeneReader and the Ion PGM in the (**A**) retrospective and the (**B**) prospective cohort. The Pearson’s correlation is indicated on the graphs. The 95% confidence interval is represented as hatched grey areas. The different underlying mutations are indicated by differently colored dots on the plot; (**C**) Density plot of tumor-cell content distribution between the retrospective (in blue) and prospective cohort (in red); (**D**) Coverage at the site of mutation in the respective genes for the GeneReader and the Ion PGM. Median with the first and third quartile is blotted in the boxes, prolonged with the 1.5× interquartile range. Outliers outside of this range are indicated as dots.

The allele frequency of the mutations between the two sequencing systems was analyzed using the Pearson’s correlation. In the retrospective cohort, correlation was very high with a *r*^2^ of 0.93 (Figure 1A). While the prospective cohort showed less correlation (*r*^2^ = 0.81), the results were still comparable between the two systems (Figure 1B). Four-times-repeated DNA Isolation and sequencing of the same sample showed high reproducibility in allele frequency between the different runs as well as high correlation between the two sequencing systems (Supplementary Materials Figure S1). For both study cohorts, samples with tumor-cell content between 10% and 90% were used. While the tumor-cell content was evenly distributed in the retrospective cohort, more samples with a higher percentage of tumor cells were included in the prospective cohort (Figure 1C). Coverage in the mutated genes differed between the sequencing systems as well as between the study cohorts (Figure 1D). Across all the study cohorts and mutated genes, the median coverage was 5.849 (Range: 420–43.084) for the GeneReader and 3.464 (Range: 314–20.522) for the Ion PGM system. The GeneReader showed a considerably higher variability of coverage. However, the coverage of the two systems did not correlate (Supplementary Materials Figure S2).

### 2.3. GeneReader and Ion PGM Show Comparable Hands-on Time but Different Total Run Times

**Figure 2:**
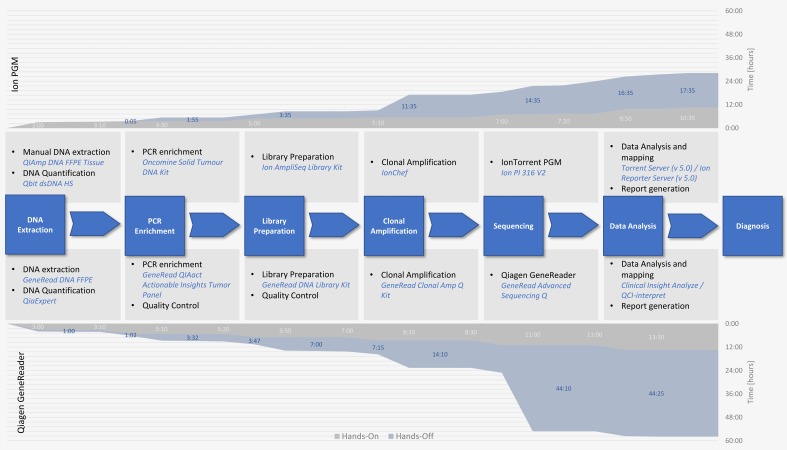
Comparison of the workflow and the different times needed for the Ion PGM workflow in contrast to the GeneReader platform. The central graph shows comparison of the different steps needed from nucleic acid extraction to diagnosis as well as required chemicals and equipment depicted in blue. Hands-on time (grey) is considerably lower for the Ion PGM (upper graph) than for the GeneReader system (lower graph). The time required is indicated in hours:minutes and highlighted in light grey. Concerning the time needed by the different devices (marked in blue), the Ion system was substantially faster than the GeneReader (marked in dark blue on the respective graphs). Therefore, the total time from start to diagnosis is longer for the GeneReader system compared to the Ion PGM (total time is indicated on the *Y*-axis on the right-hand side of each graph).

The GeneReader showed a full sample-to-insight workflow, integrating multiple Qiagen automation products for fast and reproducible sample processing of up to 40 samples per run. In contrast, the PGM workflow was developed in the laboratory and relies mainly on manual procedures with the help of the Ion Chef^®^ for automated clonal amplification and preparation of the sequencing chip and allows the sequencing of 24 samples per run. The highly automated workflow of the GeneReader has only limited influence on the hands-on time during the procedure and is still longer than the PGM workflow with approximately 13 h 30 min for the GeneReader versus 10 h 35 min for the PGM, respectively (Figure 2). The time that was required by the different machines during the processing, excluding the sequencing run, was nearly the same with approximately 14 h 25 min for the GeneReader and 14 h 35 min for the PGM (Figure 2). However, due to the different sequencing principles, the sequencing run using the Qiagen system was much longer compared to the Ion PGM system (30 h versus 3 h). Consequently, the whole workflow of the GeneReader needed approximately 5 days from sample to a final clinical report while the same result could be obtained in three and a half days using the Ion PGM workflow (Figure 2). 

## 3. Discussion

The introduction of targeted therapies into the clinical treatment of patients with advanced lung adenocarcinoma has made routine testing of tumor mutations a daily task in molecular pathology laboratories. The Qiagen GeneReader system is the first sequencing system commercially available that spans the whole workflow from nucleic acid isolation to data analysis, with the potential to replace currently used “homebrew” workflows for the genetic analysis, as previously demonstrated in comparison to Illumina MiSeq sequencing, Sanger sequencing and pyrosequencing; systems which are commonly used for genetic assessment in routine molecular pathology [15,16]. 

This is the first study to report on the comparison of the GeneReader system (Qiagen) with a widely used NGS system, the Ion PGM (Thermo Fisher Scientific, Waltham, MA, USA) in a large cohort of clinical samples from advanced lung adenocarcinoma patients. We demonstrate the general usability of the GeneReader system for routine practice in a pathology laboratory setting. The system can easily be installed and integrated into a routine pathology and only limited training of the technical personnel is necessary. The high traceability is an advantage for daily routine sequencing [17,18]. The Ion PGM is not provided with a fully integrated tracking system and thus, we had to develop our own workflow and sample tracking system in the laboratory. This timely and quite complicated process was not necessary for the GeneReader workflow as all systems and the necessary software were directly delivered and installed and worked nearly out of the box in our hands. However, the high degree of automation did not result in reduced hands-on times and faster sequencing processing. Compared to our commonly used NGS workflow using the Ion PGM system, the Qiagen system showed higher hands-on times as well as delayed sample-to-diagnosis times. Only the time needed for data analysis was considerably reduced using the GeneReader system. However, both workflows fit into a normal Monday–Friday working week and, therefore, the different sequencing times should have only a limited impact on the following clinical treatment decision. The higher sample throughput might favor the GeneReader workflow for laboratories with more samples and will consequently shorten the time from sample preparation to diagnosis. Nevertheless, a low throughput is obtained substantially faster using our accredited Ion PGM workflow. Besides the processing time, both systems clearly demonstrated that the workflow can be successfully implemented in a routine laboratory. However, the required sample input is considerably lower for the PGM. Especially when biopsies are used as the source for DNA sequencing, the total amount of DNA can be quite low and subsequently cannot be sequenced. While we did not have to face this problem during this study, the success rate at which samples can be sequenced might be considerably lower on the GeneReader in such a setting. As the GeneReader is a newly developed system, it is essential to see if there is considerable improvement in the future regarding the sample input. Depending on the individual needs, such as available laboratory space, sample throughput, the required flexibility, the initial budget and the usual source of sample, either the Ion PGM or the GeneReader may be favored. Therefore, detailed analyses of the requirements in the respective laboratory must be evaluated before favoring a certain system. Finally, the sequencing panels that are currently available differ between the two systems and thus, individual interest in specific genes remains to be considered. However, newly developed sequencing panels for the GeneReader are now commercially available and the application range will certainly be expanded. Importantly, the concordance in the case of mutation analysis and allele frequency was excellent between the two systems confirming that both can be used for high-quality sequencing in routine molecular analysis. Additionally, coverage rarely dropped below 500, allowing superior mutation assessment. Interestingly, the use of overlapping amplicons led to a higher variability in coverage with the GeneReader system. Additionally, no correlation between coverage for the two systems was obtained, indicating major technical differences. A quantitative statement about the mutations present, like amplifications of certain genes, is therefore currently not possible using the GeneReader. However, this does not interfere with the quality of the sequencing results. Failure of library preparation occurred in both workflows without favoring any system or showing superiority of one workflow over the other. 

The amount of patients harboring mutations in the prospective cohort was remarkably higher than in the retrospective cohort. This was mainly driven by the unusually high amount of *EGFR* mutations (33% vs. expected ~15%) [19]. However, the patients were selected by an external clinical tumor board and not in the laboratory and thereby enrichment of certain mutations was certainly due to the known associated clinical data. In contrast, the retrospective cohort was selected in house to also cover several patients without known mutations in the selected genes to allow the determination of false positive results which led to the differences in two study populations.

Interestingly, one sample in the prospective cohort initially showed a discordant result, where the *EGFR* p.L858R mutation was found using the GeneReader and an *EGFR* p.G719C mutation with Ion PGM analysis. However, for this patient, two different tumor blocks had to be analyzed on the two different platforms due to a low amount of input material. Both samples had a sufficient and comparable tumor-cell content of 60% and the respective mutations were confirmed after crossing the samples on the other sequencing platform. Therefore, we concluded that the discordant result is indeed due to underlying cellular heterogeneity in the tumor where different tumor clones are present, harboring different *EGFR* mutations. The *EGFR* L858R mutation, detected by the GeneReader, usually justifies an anti-*EGFR* treatment while an *EGFR* G719C mutation diagnosed by the PGM would require a more complex treatment decision [20,21]. Consequently, careful sample selection for NGS independent of the underlying system and technique used is imperative [22,23]. 

## 4. Materials and Methods 

### 4.1. Sample and DNA Isolation

Frozen and FFPE tumor specimens from various clinical sources were collected from lung adenocarcinoma patients at the Laboratory of Clinical and Experimental Pathology (LPCE) at the University Hospital of Nice. All patients had a clinical indication for molecular testing and were informed about the purpose of the molecular analysis by the treating physician. All samples were processed by the routine diagnostic pipeline of the LPCE in 2016 and 2017 and a total of 90 cases were selected. In the retrospective training cohort, 60 consecutive samples, with clinically relevant mutations in genes like *EGFR*, *KRAS* or *BRAF*, with a sufficient tumor-cell content for GeneReader analysis (>10%) were selected. Additionally, a larger amount of patients without known mutations were included. Lastly, frozen as well as FFPE samples were included in the cohort. In parallel, 30 samples in clinical routine practice were analyzed prospectively. The decision to perform sequencing was made by a clinical cancer board and was based on the clinical history of each patient. All patients provided written informed consent. The study complied with the World Medical Association Declaration of Helsinki regarding ethical conduct of research involving human subjects.

Patients were characterized for tumor-cell content, age, sex, smoking history and stage (Table 3). The relative percentage of tumor cells to other cells was estimated on a hematoxylin and eosin (H&E) stained tumor section by a board-certified molecular pathologist. An area with a tumor-cell content of >10% was designated for analysis.

DNA isolation from the macro-dissected tumor areas was carried out using the QIAamp DNA FFPE Tissue Kit (QIAGEN GmbH, Hilden, Germany) as described previously [24]. DNA isolation from frozen tumor tissue was performed using the MagNA Pure Compact Nucleic Acid Isolation Kit Large Volume (Roche Group, Inc., Tucson, AR, USA) according to the manufacturer’s instructions [25]. For the 30 prospective cases analyzed using the GeneReader, DNA was isolated using the GeneRead™ DNA FFPE Kit (QIAGEN GmbH, Hilden, Germany) according to the manufacturer’s protocol. The nucleic acid concentration was measured with the Qubit dsDNA HS Assay kits on the Qubit 2.0 Fluorometer (Thermo Fisher Scientific). Prospective DNA samples were quantified using the QIAxpert.

### 4.2. Ion PGM Assay and Sequencing

A total of 10 ng of DNA was processed using the Ion Oncomine™ Panel using the Ion AmpliSeq™ Library Kit™ (Ion PGM™, Thermo Fisher Scientific, Illkirch-Graffenstaden, France) [26]. This panel covers multiple exons in 22 genes with 92 amplicons, each covering an individual region in the target genome (Table 1). Samples were barcoded and libraries from each sample were pooled at 20 pM concentrations. Ion chef was used for automated template preparation, enrichment of ion spheres and chip loading on an Ion PI™ 316 Chip v2 for sequencing using the Ion Personal Genome Machine^®^ (Ion PGM™, ThermoFisher Scientific, Waltham, MA, USA). Results were analyzed using the Variant caller by aligning the reads to the hg19 reference genome, calling the variants, and generating an interactive report for visualization and quality control. Data analysis was performed using Torrent Server™ (v 5.0) and Ion Reporter™ Server hosting informatic tools (Ion Reporter™ Software v5.0) for variant analysis, filtering and annotations. The sequencing run was considered successful and the quality adequate when the following quality metrics were met: (1) mapped reads ≥300,000; (2) average base coverage depth ≥300; (3) amplicons with at least 300 reads: ≥99%; (4) no strand bias: ≥95%; (5) amplicons read end-to-end: ≥99%. Integrative Genome Viewer (IGV) was utilized for visualization. The cut-off was set at 300× coverage and minimum 5% allelic frequency. Mutation detection sensitivity of each Ion PGM™ run was determined by AcroMetrix™ Oncology Hotspot Control for heterozygous mutations in seven different genes: *EGFR* (ENSG00000146648), *KRAS* (ENSG00000133703), *BRAF* (ENSG00000157764), *NRAS* (ENSG00000213281), *ALK* (ENSG00000171094), *PIK3CA* (ENSG00000121879) and *ERBB2* (ENSG00000141736). 

### 4.3. GeneReader Assay and Sequencing

In total, 40 ng of each DNA sample were used as template for the QIAGEN Actionable Insight Tumor Panel according to the manufacturer’s instructions. The 330 amplicons in the panel cover multiple exons in 12 genes (Table 1). The amplicons are designed to overlap each other, thereby multiple amplicons are spanning the same region. Libraries were prepared using the QIAGEN GeneRead DNA Library Kit and an automated protocol on a QIAcube. PCR-enriched DNA and GeneRead libraries were qualified and quantified using a QIAGEN QIAxcel Advanced System. Emulsion PCR and bead enrichment steps were carried out using the GeneRead Clonal Amp Q Kit on a GeneRead QIAcube. Following clonal amplification, amplicon libraries were sequenced using the QIAGEN GeneRead Sequencing Q Kit and after an upgrade during the testing period, the GeneRead Advanced Sequencing Q Add-On on a GeneReader instrument (all protocols available on http://www.qiagen.com). QIAGEN Clinical Insight Analyze (QCI-A) software performed the secondary analysis of FASTQ reads generated by the GeneReader. Variants were imported into the QCI-interpret (QCI-I) web interface for data interpretation and report generation. 

### 4.4. Data Analysis

Data was analyzed using R software for statistical computing [27] and graphs were generated using the ggplot2 package for R [28]. Correlation coefficients for the allele frequency at the site of mutation between the two systems were calculated according to Pearson.

## 5. Conclusions

Taken together, our results demonstrated that the sequencing performance of both the Ion PGM in combination with the Oncomine targeted sequencing panel and the Qiagen GeneReader with the Actionable Insights targeted sequencing panel justifies their installation and use in a routine molecular pathology laboratory. For a low sample input, the workflow on the Ion PGM is quite faster with a lower hands-on time. However, this might vary in settings where additional throughputs are required due to the considerably higher processing capacity of the GeneReader. Moreover, it is offered in a pay-per-sample model and thus might be more suitable in clinics where the high initial investment for the installation of an NGS system usually presents an insurmountable obstacle. Thus, the newly developed Qiagen GeneReader system clearly demonstrates its performance in routine NGS analysis of lung cancer patients but to finally decide between the two systems, the individual requirements as well as the future demand of sample throughput must be strongly considered. 

## Figures and Tables

**Table 1:** Comparison of Ion Torrent Oncomine and GeneReader Actionable Insights targeted sequencing panels.

	Ion Oncomine Panel on PGM	QIAGEN Actionable Insight Tumor Panel on GeneReader
**Panel Size**	22 genes; 11 kb	12 genes; 16.7 kb
**Gene list ^#^**	*EGFR*, *ALK*, *ERBB2*, *ERBB4*, *FGFR1*, *FGFR2*, *FGFR3*, *MET*, *DDR2*, *KRAS*, *PIK3CA*, *BRAF*, *AKT1*, *PTEN*, *NRAS*, *MAP2K1*, *STK11*, *NOTCH1*, *CTNNB1*, *SMAD4*, *FBXW7*, *TP53*	*KRAS*, *NRAS*, *KIT*, *BRAF*, *PDGFRA*, *ALK*, *EGFR*, *ERBB2*, *PIK3CA*, *ERBB3*, *ESR1*, *RAF1*
**Amplicons**	92	330
**Variant allele fraction detection limit**	5%	5%
**DNA amount**	10 ng	40 ng
**Minimal base coverage depth**	>300X ^ǂ^	Not specified by the manufacturer
**Label**	CE IVD	RUO

IVD in vitro diagnostic use, RUO Research-use only, ^#^ overlapping genes between the two sequencing panels are underlined, ^ǂ^ as authorized by the French National Cancer Institute (INCa) and accredited to ISO 15189 by COFRAC [12,14].

**Table 2:** Description of the mutational status of the 90 patients with advanced non-small cell lung cancer included in the study.

Mutation Status	Retrospective Cohort [*N* = 60]	Prospective Cohort [*N* = 30]
**No detected mutations**	25 (42%)	5 (17%)
**Mutations detected in**	33 (55%)	25 (83%)
***EGFR***	9 (15%)	8 (27%)
***KRAS***	20 (33%)	13 (43%)
***BRAF***	3 (5%)	2 (7%)
**Double Mutations**		
*EGFR del19 & KRAS p.G12D*	1 (2%)	na
*EGFR p.V774M & p.L861Q*	na	1 (3%)
*EGFR p.L858R & p.G719C*	na	1 (3%)
**Failed analysis**	2 (3%)	na

**Table 3:** Description of the patient cohort.

	Retrospective Cohort	Prospective Cohort	Total
**N**	60 (67%)	30 (33%)	90 (100%)
**Tumor Tissue**			
Frozen	12 (20%)	na	12 (13%)
FFPE	48 (80%)	30 (100%)	78 (87 %)
**Age**			
Median (range)	67 (50–90)	69 (47–86)	68 (47–90)
**Sex**			
Female	24 (40%)	13 (43%)	37 (41%)
Male	36 (60%)	17 (57%)	53 (59%)
**Smoking**			
Current smoker	24 (40%)	5 (17%)	29 (32%)
Former smoker	18 (30%)	8 (27%)	26 (29%)
Non-smoker	5 (8%)	6 (20%)	11 (12%)
Unknown	13 (22%)	11 (37%)	24 (27%)
**Tumor-cell content Median (range) [%]**	50 (10–90)	70 (10–90)	60 (10–90)
**TNM stage**			
II	11 (18%)	7 (23%)	18 (20%)
III	11 (18%)	3 (10%)	14 (16%)
IV	38 (63%)	20 (67%)	58 (64%)

## Supplementary Materials

The following are available online at http://www.mdpi.com/2072-6694/10/4/88/s1, Figure S1: For one Sample, DNA Isolation and Sequencing was repeated 4 times. Allele Frequency of the *KRAS* p.Q61H mutation after repeated sequencing using the GeneReader and Ion PGM was determined to assess reproducibility of the assay; Figure S2: Comparison of the coverage between the GeneReader and the PGM in the **A** retrospective and the **B** prospective cohort. Coverage at the site of the mutation in the PGM is plotted against the coverage at the site of mutation for the GeneReader. The Pearson’s correlation is indicated on the graphs. The 95% confidence interval is represented as hatched grey areas. Different mutations are represented in different colored dots as mentioned on the graph; Table S1: Description of Patient cohort; Table S2: Comparison of detected alterations between the two sequencing systems by type of alteration.
