# Supplementary material for: Use of the Ion PGM and the GeneReader NGS Systems in Daily Routine Practice for Advanced Lung Adenocarcinoma Patients: A Practical Point of View Reporting a Comparative Study and Assessment of 90 Patients
Source: Cancers (Basel). 2018 Mar 21;10(4):88. doi: 10.3390/cancers10040088 (PMC5923343; doi:10.3390/cancers10040088)
Supplement: Supplementary file 1 [file cancers-10-00088-s001.pdf]

Use of the Ion PGM and the GeneReader NGS systems in daily routine practice for advanced lung adenocarcinoma patients. A practical point of view reporting a comparative study and assessment of 90 patients

Simon Heeke, Véronique Hofman, Elodie Long-Mira, Virginie Lespinet, Salomé Lalvée, Olivier Bordone, Camille Ribeyre, Virginie Tanga, Jonathan Benzaquen, Sylvie Leroy, Charlotte Cohen, Jérôme Mouroux, Charles Hugo Marquette, Marius Ilié and Paul Hofman

Supplementary Material

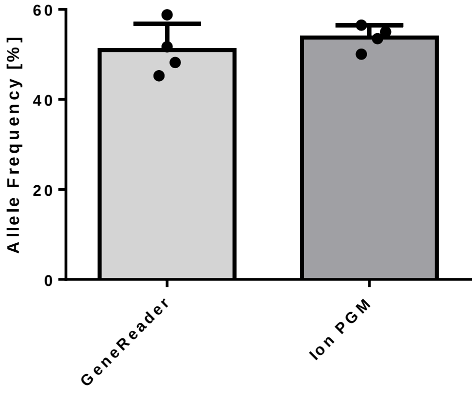

Figure S1. For one Sample, DNA Isolation and Sequencing was repeated 4 times. Allele Frequency of the *KRAS* p.Q61H mutation after repeated sequencing using the GeneReader and Ion PGM was determined to assess reproducibility of the assay.

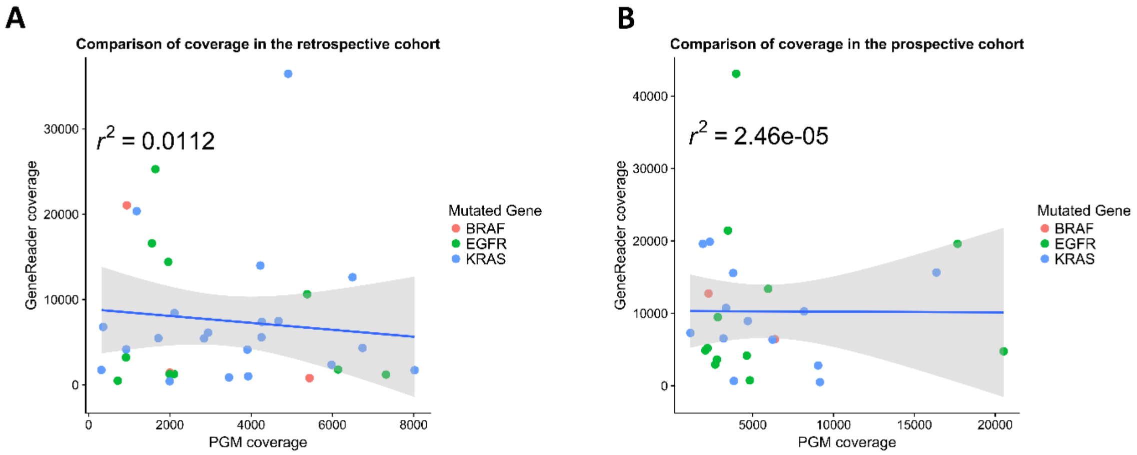

Figure S2. Comparison of the coverage between the GeneReader and the PGM in the **A** retrospective and the **B** prospective cohort. Coverage at the site of the mutation in the PGM is plotted against the coverage at the site of mutation for the GeneReader. The Pearson's correlation is indicated on the graphs. The 95% confidence interval is represented as hatched grey areas. Different mutations are represented in different colored dots as mentioned on the graph.

Table S1. Description of patient cohort.

Prospective Cohort				
**Patient**	**Tumor sample origin**	**Sample Preservation**	**Tumor cell content [%]**	**Mutation**
1	Bronchoscopic biopsy	FFPE	40	No Mutation detected
2	Biopsy under video mediastinoscopy	FFPE	70	*EGFR* p.E746_A750del
3	Surgical Specimen	FFPE	80	No Mutation detected
4	Thoracoscopic biopsy	FFPE	60	*EGFR* p.A767_V769dup
5	Surgical Specimen	FFPE	80	*EGFR* p.L858R
6	Surgical Specimen	FFPE	80	*EGFR* p.L861Q / *EGFR* p.V774M
7	Bronchoscopic biopsy	FFPE	20	*BRAF* p.G469A
8	Surgical Specimen	FFPE	80	*EGFR* p.E746_A750del
9	Surgical Specimen	FFPE	90	*BRAF* p.V600E
10	Surgical Specimen	FFPE	10	*KRAS* p.G12D
11	Surgical Specimen	FFPE	60	No Mutation detected
12	Surgical Specimen	FFPE	40	No Mutation detected
13	Surgical Specimen	FFPE	50	*KRAS* p.G12V
14	Surgical Specimen	FFPE	90	*KRAS* p.G12C
15	Surgical Specimen	FFPE	30	*KRAS* p.G12D
16	Surgical Specimen	FFPE	70	*KRAS* p.G12D
17	Surgical Specimen	FFPE	50	*KRAS* p.G12V
18	Surgical Specimen	FFPE	90	*KRAS* p.G12C
19	Surgical Specimen	FFPE	80	*KRAS* p.G12C
20	Surgical Specimen	FFPE	90	*KRAS* p.G12C
21	Surgical Specimen	FFPE	60	*EGFR* p.L858R / *EGFR* p.G719C
22	Surgical Specimen	FFPE	70	*EGFR* p.L858R
23	Surgical Specimen	FFPE	70	*KRAS* p.G12C
24	Surgical Specimen	FFPE	80	No Mutation detected
25	Surgical Specimen	FFPE	70	*EGFR* p.E746_A750del
26	Surgical Specimen	FFPE	80	*KRAS* p.G12C
27	Surgical Specimen	FFPE	30	*EGFR* p.L858R
28	Surgical Specimen	FFPE	60	*EGFR* p.E746_A750del
29	Surgical Specimen	FFPE	80	*KRAS* p.G12C
30	Thoracoscopic biopsy	FFPE	20	*KRAS* p.G12D

Retrospective Cohort				
**Patient**	**Tumor sample origine**	**Sample Preservation**	**Tumor cell content [%]**	**Mutation**
1	Biopsy under video mediastinoscopy	frozen	50	*KRAS* p.G12A
2	Surgical Specimen	frozen	30	No Mutation detected
3	Surgical Specimen	frozen	50	No Mutation detected
4	Thoracoscopic biopsy	FFPE	30	*EGFR* p.L858R

5	Surgical Specimen	frozen	60	*KRAS* p.A146T
6	Surgical Specimen	frozen	60	No Mutation detected
7	Surgical Specimen	FFPE	30	*EGFR* p.G719A
8	Biopsy under video mediastinoscopy	FFPE	60	*BRAF* p.V600E
9	Thoracoscopic biopsy	FFPE	20	No Mutation detected
10	Surgical Specimen	frozen	70	No Mutation detected
11	Fine needle biopsy	FFPE	70	*EGFR* p.L858R
12	Thoracoscopic biopsy	FFPE	40	*KRAS* p.G12D<br>(library preparation failed on PGM)
13	Surgical Specimen	FFPE	60	*KRAS* p.G12C
14	Surgical Specimen	FFPE	60	No Mutation detected
15	Biopsy under video mediastinoscopy	FFPE	50	*KRAS* p.G12C
16	Fine needle biopsy	FFPE	20	No Mutation detected
17	Biopsy under video mediastinoscopy	FFPE	50	No Mutation detected
18	Biopsy under video mediastinoscopy	FFPE	20	*KRAS* p.G12V
19	Surgical Specimen	frozen	80	No Mutation detected
20	Surgical Specimen	FFPE	40	No Mutation detected
21	Surgical Specimen	FFPE	50	No Mutation detected
22	Surgical Specimen	FFPE	20	No Mutation detected
23	Surgical Specimen	FFPE	80	*EGFR* E746_A750del
24	Surgical Specimen	FFPE	40	*KRAS* p.G12C
25	Biopsy under video mediastinoscopy	FFPE	30	No Mutation detected
26	Thoracoscopic biopsy	FFPE	80	*BRAF* p.V600E
27	Surgical Specimen	FFPE	70	*EGFR* p.S768I
28	Fine needle biopsy	FFPE	70	No Mutation detected
29	Surgical Specimen	FFPE	90	*BRAF* p.G469A
30	Bronchoscopic biopsy	FFPE	20	No Mutation detected
31	Bronchoscopic biopsy	FFPE	20	*EGFR* p.E746_A750del
32	Bronchoscopic biopsy	FFPE	80	*EGFR* p.E746_A750del
33	Surgical Specimen	FFPE	15	No Mutation detected
34	Biopsy under video mediastinoscopy	FFPE	50	No Mutation detected
35	Surgical Specimen	FFPE	70	No Mutation detected
36	Surgical Specimen	FFPE	30	No Mutation detected
37	Surgical Specimen	FFPE	30	No Mutation detected
38	Bronchoscopic biopsy	frozen	40	*KRAS* p.G12C
39	Biopsy under video mediastinoscopy	FFPE	80	*KRAS* p.G12D
40	Surgical Specimen	FFPE	80	*KRAS* p.Q61H
41	Biopsy under video mediastinoscopy	frozen	70	*KRAS* p.G13C
42	Surgical Specimen	FFPE	30	No Mutation detected

43	Biopsy under video mediastinoscopy	FFPE	50	No Mutation detected
44	Surgical Specimen	FFPE	40	*KRAS* p.G12C
45	Bronchoscopic biopsy	FFPE	50	*KRAS* p.G12V
46	Bronchoscopic biopsy	FFPE	30	*KRAS* p.G12A
47	Surgical Specimen	FFPE	70	*EGFR* p.E746_T751>A
48	Thoracoscopic biopsy	frozen	30	*KRAS* p.G12C
49	Surgical Specimen	FFPE	90	No Mutation detected
50	Surgical Specimen	FFPE	50	*EGFR* p.E746_E751del / *KRAS* p.G12D
51	Surgical Specimen	frozen	50	No Mutation detected
52	Surgical Specimen	FFPE	60	No Mutation detected
53	Surgical Specimen	FFPE	70	*KRAS* p.G13C
54	Surgical Specimen	FFPE	90	*KRAS* p.Q61H
55	Biopsy under video mediastinoscopy	FFPE	70	*KRAS* p.G12C
56	Biopsy under video mediastinoscopy	FFPE	90	*KRAS* p.G12C
57	Surgical Specimen	frozen	50	*EGFR* p.L747_T751
58	Bronchoscopic biopsy	FFPE	10	*KRAS* p.G12V
59	Surgical Specimen	FFPE	80	*KRAS* p.G12V
60	Bronchoscopic biopsy	FFPE	30	*EGFR* p.L747_A750>P (library preparation failed on GeneReader)

Table S2: Comparison of detected alterations between the two sequencing systems by type of alteration

Alteration	Ion PGM mutations	GeneReader mutations	False positives	False negatives
Missense Mutation	50	50	0	0
Deletion	9	9	0	0
Complex Deletion	1	1	0	0
Insertion	1	1	0	0
